# Zim CHIC: A cohort study of immune changes in the female genital tract associated with initiation and use of contraceptives

**DOI:** 10.1111/aji.13287

**Published:** 2020-06-25

**Authors:** Sharon L. Achilles, Leslie A. Meyn, Felix G. Mhlanga, Allen T. Matubu, Kevin A. Stoner, May A. Beamer, Zvavahera M. Chirenje, Sharon L. Hillier

**Affiliations:** ^1^ Department of Obstetrics, Gynecology, and Reproductive Sciences and Center for Family Planning Research University of Pittsburgh School of Medicine Pittsburgh PA USA; ^2^ Magee‐Womens Research Institute Pittsburgh PA USA; ^3^ Department of Obstetrics and Gynaecology University of Zimbabwe College of Health Science Harare Zimbabwe

**Keywords:** biomarkers, CCR5, contraception, copper, medroxyprogesterone acetate, receptors

## Abstract

**Problem:**

Contraceptive hormones are systemically active, potent, and likely to invoke biological responses other than known fertility regulation impacts. We hypothesized that initiation of depot medroxyprogesterone acetate (DMPA) would increase genital HIV‐target‐cells and soluble immune mediators compared with baseline and initiation of other contraceptive methods.

**Method of Study:**

We collected cervical cytobrushes and cervicovaginal fluid from healthy Zimbabwean women aged 18‐34 to assess immune cell populations, cytokines, and innate anti‐HIV activity at baseline and after 30, 90, and 180 days use of DMPA (n = 38), norethisterone enanthate (n = 41), medroxyprogesterone acetate/estradiol cypionate (n = 36), levonorgestrel implant (n = 43), etonogestrel implant (n = 47), or copper intrauterine device (Cu‐IUD) (n = 45). Cells were quantified by flow cytometry, cytokines were detected by multiplex assays, and innate anti‐HIV activity was assessed by in vitro HIV challenge.

**Results:**

Compared to baseline, the number of cervical HIV target cells (#CD4 cells *P* < .04 and #CD11c cells *P* < .04), the concentration of the inflammatory cytokine IL‐1β (*P* < .01), and the innate in vitro anti‐HIV activity (*P* < .001) significantly decreased following DMPA initiation. In Cu‐IUD users, genital HIV target cells increased (#CD4 cells *P* < .001, #CD4CCR5 cells *P* = .02, #CD4CD69 cells *P* < .001, #CD8CD69 *P* = .01, and #CD11c cells *P* = .003) at day 30 and resolved by day 180. IFN‐γ (*P* < .001), IL‐1β (*P* < .001), IL‐6 (*P* < .001), IL‐8 (*P* < .001), IL‐10 (*P* < .01), and RANTES (*P* < .001) were also significantly increased at day 30. Minimal alterations were observed following initiation of subdermal implantable contraceptives.

**Conclusions:**

This head‐to‐head study compared six contraceptives and found increased HIV target cells and cervical inflammation temporally associated with Cu‐IUD initiation. Use of hormonal contraception, including DMPA, did not increase cervical HIV target cells or inflammation.

Clinical Trial Number: NCT02038335

## 1 INTRODUCTION

Globally, approximately 60% of all reproductive‐aged women use modern contraception, including ~256 million intrauterine device users and ~93 million injectable contraceptive users.^1^, ^2^ Observational studies evaluating HIV acquisition risk associated with contraceptive use suggest that use of depot medroxyprogesterone acetate (DMPA) may be associated with increased risk,^3^ however, no substantial difference in HIV risk among DMPA, copper intrauterine device (Cu‐IUD), and levonorgestrel implant (LNG‐I) users was demonstrated in a recent randomized trial.^4^

Increased susceptibility to HIV has been reported in women having vaginal dysbiosis^5^, ^6^, ^7^, ^8^, ^9^ and increased genital inflammation.^10^, ^11^, ^12^ Since immune cells are primary targets for HIV transmission, there is biologic plausibility for increased HIV acquisition risk associated with increased local inflammation, target cell density, dysbiosis, and decreased innate anti‐HIV activity, any of which may be impacted by contraceptive use.

Contraceptives vary by composition (hormonal or non‐hormonal and types of hormones), route of delivery (oral, transdermal, subdermal, injectable, vaginal, and intrauterine), and resultant drug pharmacokinetics and pharmacodynamics. This heterogeneity, compounded by the phenomenon of frequent contraceptive switching, has challenged careful study of biological changes in women.

We aimed to evaluate the impact of contraceptive initiation and use on genital tract immune cells, soluble mediators of inflammation, and innate anti‐HIV activity, all of which could alter HIV susceptibility. We evaluated CD4 and CD8 T‐cells, CD11c^+^ antigen‐presenting cells (APCs), as well as cellular expression of CCR5 (HIV co‐receptor) and CD69 (activation marker), all implicated as necessary or important for acquisition of HIV in women.^13^, ^14^, ^15^ We hypothesized that initiation and use of DMPA would increase genital tract HIV target cells and soluble immune mediators compared with baseline and to use of non‐injectable contraceptive methods.

## 2 METHODS

We performed a parallel longitudinal cohort study (ClinicalTrials.gov number: NCT02038335) of healthy Zimbabwean women initiating contraception with an injectable (DMPA, Net‐En, MPA/EC), an implant (LNG‐I or ENG‐I), or a Cu‐IUD (copper T380A IUD). The study was designed to assess genital tract changes in HIV target cells, immunoregulatory cytokines, and innate anti‐HIV activity compared to baseline with each woman serving as her own control after 30, 90, and 180 days of contraceptive use relative to quantified serum progestogen concentrations. The primary objective was change from baseline in lower genital tract HIV target cells after 90‐days of continuous contraceptive use. At each visit, we quantified serum progesterone, levonorgestrel (LNG), etonogestrel (ENG), norethindrone (NET), and medroxyprogesterone acetate (MPA) concentrations, covering the spectrum of regionally available progestins as previously described.^16^, ^17^ Participants having confirmed non‐study hormone use at enrollment or follow‐up were not included in this analysis.

We calculated a sample size of 35 women in each group would provide 80% power to detect a 20% change in HIV target cell populations, based on a paired samples *t* test and a mean CD3^+^CD4^+^CCR5^+^ cell recovery from cervical cytobrush at enrollment observed in a prior study.^18^, ^19^ To account for the use of non‐parametric statistical methods and loss to follow‐up, we planned to enroll 50 women per contraceptive group.

All participants were enrolled at Spilhaus Family Planning Centre in Harare, Zimbabwe and signed informed consent before study participation. The protocol was approved by the University of Pittsburgh Institutional Review Board and The Medical Research Council of Zimbabwe.

### 2.1 Study population

Eligible women were healthy, aged 18‐34 years, HIV negative, non‐pregnant and reported regular menstrual cycles. Women were excluded if within 30 days of enrollment they: (a) self‐reported any hormonal or intrauterine contraceptive use; (b) underwent any genital tract procedure including biopsy; (c) were diagnosed with any urogenital tract infection; (d) used any oral or vaginal antibiotics, oral or vaginal steroids, or any vaginal product or device except tampons and condoms. Women were also excluded if they used DMPA within 10 months of enrollment, were pregnant or breastfeeding within 60 days of enrollment, had a new sexual partner within 90 days of enrollment, or had a prior hysterectomy or malignancy.

Screening included urine pregnancy testing, 2 rapid HIV screening tests, and collection of cervicovaginal fluid by swab to biologically confirm absence of genital tract infections as previously described.^16^, ^20^ Eligible participants then presented for enrollment during the follicular phase of menses (self‐reported day 1‐14). At enrollment and every follow‐up visit, participants were free of vaginal bleeding and had refrained from vaginal and anal intercourse for ≥48 hours (self‐report). Contraceptives were administered immediately following sample collection by a study clinician per standard practice at enrollment and as clinically indicated at follow‐up visits, with strict adherence to the varied dosing schedules of three injectable contraceptives: every 30, 60 and 90 days for MPA/EC, Net‐En, and DMPA, respectively. Laboratory personnel in Zimbabwe and the United States was masked to participant clinical status, including the contraceptive group.

### 2.2 Sample collection and processing

Peripheral blood mononuclear cells (PBMCs), endocervical cytobrush, neat vaginal fluid, and cervicovaginal lavage samples were collected at enrollment and at 30, 90, and 180 days, placed on ice and received by the Zimbabwean laboratory within 2 hours of collection. Blood and genital tract samples for hormonal and flow cytometric analyses were processed as previously described.^16^, ^17^ Peripheral blood mononuclear cells (PBMCs) were obtained by venipuncture using Vacutainer^®^ CPT tubes (BD Biosciences). Tubes were centrifuged at 1300 *g* for 30 minutes with the brake off. The cell layer was removed, washed twice with DPBS (Corning) by centrifugation at 400 *g*, resuspended in 2 mL of DPBS, and viable cells were counted using Trypan blue (Sigma‐Aldrich) exclusion.^21^ Endocervical specimens were obtained by inserting a cytobrush (Cooper Surgical, Trumbull, CT) into the cervical os, rotating 360°, and placing in 4 mL RPMI‐1640 medium supplemented with 25 mmol/L HEPES, L‐glutamine, and 10% fetal bovine serum (tRPMI). In the laboratory, mucus and cells were recovered by scraping the cytobrush on a polypropylene conical tube while rinsing with tRPMI until no visible material remained. The suspension was filtered through a 70 µm mesh filter (BD Biosciences) using a rubber‐tipped plunger from a 5cc syringe while rinsing with tRPMI to push all material through the filter. We centrifuged the sample at 400 *g* for 10 minutes, decanted the supernatant, resuspended the pellet in 1 mL DPBS, and counted viable cells by Trypan blue exclusion. Neat vaginal fluid was collected from the posterior fornix with four Dacron swabs, one was used for bacterial vaginosis assessment according to Nugent criteria,^22^ and the others were immediately placed in sterile cryovials. Cervicovaginal lavage (CVL) was collected from the posterior fornix after rinsing the vagina and cervix with 10 mL sterile PBS for 2 minutes. Swabs and CVL were transported to the laboratory on ice and stored at −80°C.

### 2.3 Flow cytometry

Cell suspensions were adjusted to 1 × 10^6^ cells/mL with DPBS and stained per manufacturer's instructions using LIVE/DEAD^®^ Fixable Near IR (Thermo Fisher Scientific). Cells were washed with 1 mL flow cytometry staining buffer (FACS) (Thermo Fisher), centrifuged (400 *g* for 5 minutes) and stained with titrated fluorochrome‐conjugated antibodies (BD Biosciences) specific for: CD3(PerCP), CD8(AmCyan), CD4(FITC), CD195(CCR5)(PE‐Cy^™^7), CD196(CCR6)(AlexaFlour^®^647), CD69(PE), and CD11c(V450). Samples were volume adjusted (100 µL with FACS), incubated (25 minutes, room temperature, protected from light), washed (1 mL FACS), centrifuged (400 *g* for 5 minutes), washed (2 mL cold RBC Lysis Buffer (ThermoFisher) for 2 minutes), diluted (2 mL of cold DPBS), centrifuged (400 *g* for 5 minutes), decanted, and resuspended (200 µL of FACS). The stained fresh samples were temporarily stored at 4°C until flow cytometric analysis was conducted. No samples were frozen prior to flow cytometric analysis.

Cell populations were analyzed using a FACS Canto‐II flow cytometer (BD Biosciences) and FlowJo software v.10.0.5 (TreeStar). Single color compensation was applied specific for each fluorochrome‐conjugate. T‐cell populations were identified using forward and side scatter. Fluorescent‐minus‐one (FMO) controls were utilized to define gate positions. Application settings were held constant for the entirety of the study and used to control for fluctuations in flow cytometer performance and lot variations in control beads. Two advanced flow cytometrists (KAS and MAB) masked to contraceptive group, independently reviewed and agreed upon gating parameters for each sample.

### 2.4 Soluble mediators

We quantified interferon‐gamma (IFNg), interleukin 1 beta (IL‐1b), interleukin 6 (IL6), interleukin 8 (IL8), interleukin 10 (IL10), and RANTES in vaginal fluid by adding 1 mL PBS to each thawed vaginal swab and agitating at low speed and room temperature for 2 hours. We filtered the sample using Costar^®^ Spin‐X Centrifuge Tube Filter (Corning), tested the eluent in duplicate for cytokines using magnetic bead kits (EMD Millipore) according to manufacturer instructions, and analyzed with MAGPIX and xPONENT^®^ 4.2 software (Luminex).

### 2.5 Innate in vitro anti‐HIV activity in vaginal fluid

We treated TZM‐bl cells with CVL fluid or control buffer prior to challenge with HIV‐1_Bal_ as previously described^23^ to assess innate anti‐HIV‐1 activity in vitro. Results are reported as percent HIV suppression associated with CVL relative to the buffer‐only control.

### 2.6 Statistical analysis

Statistical analysis was performed using SPSS^®^ Statistical software version 23.0 (IBM Corporation), and statistical tests were evaluated at the 2‐sided .05 significance level. Cell quantities were reported as “cells per cytobrush.” For each participant, we assessed change at follow‐up compared to the baseline visit (untreated control). Differences in enrollment characteristics between the groups were assessed using one‐way analysis of variance, Kruskal‐Wallis, and Fisher's exact tests, where appropriate. Differences in levels of expression from baseline to follow‐up were evaluated using paired Wilcoxon signed‐rank tests. Given that we previously demonstrated an increase in BV after initiation of Cu‐IUD in this cohort,^20^ to understand if BV was driving any observed changes, we additionally analyzed women who initiated Cu‐IUD limited to those who were negative for BV at all visits. Non‐parametric tests were used to evaluate expression of immune cells, soluble mediators, and innate in vitro anti‐HIV activity as graphical displays of the data indicated that they did not follow a gaussian distribution. Within each contraceptive method (DMPA, Net‐En, MPA/EC, LNG‐I, ENG‐I, Cu‐IUD), compartment (local vs systemic) and follow‐up visit (30‐, 90‐, 180‐day), seven hypotheses were tested for immune cell changes (number of cells and percent parent population of CD3^+^CD4^+^, CD3^+^CD4^+^CCR5^+^, CD3^+^CD4^+^CD69^+^, CD3^+^CD8^+^, CD3^+^CD8^+^CCR5^+^, CD3^+^CD8^+^CD69^+^, CD3^+^CD11c^+^) and seven hypotheses were tested for changes in soluble mediators (IFNg, IL‐1b, IL6, IL8, IL10, RANTES, innate in vitro anti‐HIV activity). To control the type I error for the analyses of multiple endpoints, the Holm‐Bonferroni sequential correction^24^ was used to adjust the *P* values presented in the tables. Outcome data are reported as medians with corresponding interquartile ranges and percent change from baseline.

## 3 RESULTS

### 3.1 Participant enrollment and demographic characteristics

**Figure 1:**
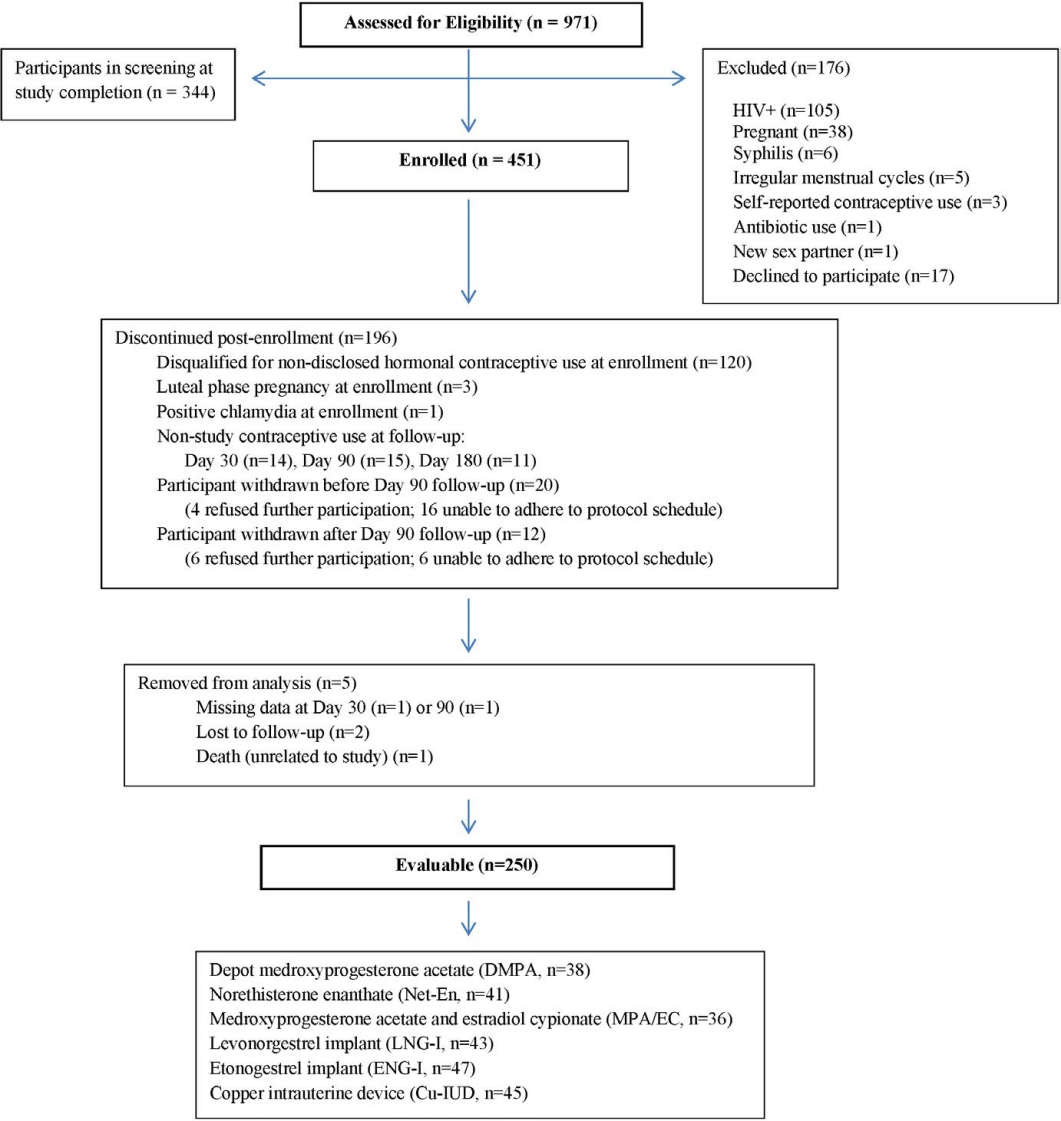
Diagram of participant flow from eligibility assessment to final categorization

Figure 1 is a flow diagram of all screened participants. Based on ultra‐high‐performance liquid chromatography tandem mass spectrometry (UPLC/MS/MS), 327 (73%) enrolled women were free of exogenous progestins at enrollment and of these, 276 (84%) were in the follicular phase of menses (progesterone <1000 pg/mL) and 51 (16%) were not (progesterone ≥1000 pg/mL), with median progesterone values of 41 (IQR 12.5, 64.8) and 4758 (IQR 2430, 8245) pg/mL, respectively. As previously reported, phase of the menstrual cycle (follicular vs non‐follicular) did not significantly impact BV prevalence (*P *> .09).^20^ Of the enrolled participants, 250 (55%) were evaluable (Figure 1) and initiated contraception with: DMPA (n = 38), norethisterone enanthate (Net‐En) (n = 41), medroxyprogesterone acetate/estradiol cypionate (MPA/EC) (n = 36), LNG‐I (n = 43), etonogestrel implant (ENG‐I) (n = 47), and Cu‐IUD (n = 45). Follow‐up visits were conducted at day 30 (median day 29, interquartile range [IQR] 28‐32), day 90 (median day 87.5, IQR 86‐91), and day 180 (median day 177, IQR 175‐182) after initiation and continuous contraceptive use.

Evaluable participants were less likely than non‐evaluable participants to select DMPA (15% vs 25%) and more likely to select ENG‐I (19% vs 9%) and Cu‐IUD (18% vs 11%) (*P* = .001). Evaluable participants were older (27 ± 4 vs 26 ± 4 years, *P* = .02) and otherwise did not differ in demographic or sexual behavioral features compared with non‐evaluable participants.

Among evaluable participants in self‐selected contraceptive groups, women opting for DMPA had lower BMI, whereas women opting for Cu‐IUD had less frequent intercourse and were less likely married/living with a partner (Table 1). Of evaluable participants, 245/250 (98%) reported sexual partners at enrollment, with no difference by group. Frequency of sexual intercourse was similar between the groups throughout the study with the exception of increased sexual frequency at the 180‐day visit among injectable contraceptive users compared to women using implants or Cu‐IUD (*P* = .04). Five of the 451 total enrolled women seroconverted to HIV+ over 199 women‐years of follow‐up (2.5% seroconversion/y) and were using the following contraceptives: ENG‐I (2), MPA/EC (1), DMPA (1), and Cu‐IUD (1). Of the 250 evaluable participants, two seroconverted while using ENG‐I (1) and DMPA (1).

**Table 1:** Demographic characteristics of evaluable participants by self‐selected contraceptive group

	Evaluable participants						*P*‐value^†^
	DMPA (n = 38)	Net‐En (n = 41)	MPA/EC (n = 36)	LNG‐I (n = 43)	ENG‐I (n = 47)	Cu‐IUD (n = 45)	
Age, y	26.4 ± 4.3	26.4 ± 3.8	28.4 ± 4.4	26.6 ± 4.1	27.5 ± 3.7	27.8 ± 4.4	.14^‡^
Gravidity (median, IQR)	2 (1.75, 3)	2 (1, 2)	2 (2, 3)	2 (1, 3)	2 (1, 3)	2 (1, 3)	.13^§^
Parity (median, IQR)	2 (1.75, 3)	2 (1, 2)	2 (2, 2.75)	2 (1, 2)	2 (1, 3)	2 (1, 2.5)	
Body mass index (kg/m^2^)	23.2 ± 3.3	24.4 ± 4.4	26.9 ± 5.8	26.4 ± 4.2	25.3 ± 4.5	26.6 ± 5.2	.002^‡^
Ethnicity							
Shona	36 (94.7%)	40 (97.6%)	34 (94.4%)	39 (90.7%)	44 (93.6%)	41 (91.1%)	.83
Other	2 (5.3%)	1 (2.4%)	2 (5.6%)	4 (9.3%)	3 (6.4%)	4 (8.9%)	
Marital status							
Married	33 (86.8%)	36 (87.8%)	34 (94.4%)	39 (90.7%)	40 (85.1%)	30 (66.7%)	.017
Not married	5 (13.2%)	5 (12.2%)	2 (5.6%)	4 (9.3%)	7 (14.9%)	15 (23.3%)	
Partner status							
Lives with partner	33 (86.8%)	36 (87.8%)	33 (91.7%)	38 (88.4%)	41 (87.2%)	29 (64.4%)	.019
Does not live with partner	5 (13.2%)	5 (12.2%)	3 (8.3%)	5 (11.6%)	6 (12.8%)	16 (35.6%)	
Education							
Primary or less	8 (21.1%)	1 (2.4%)	3 (8.3%)	3 (7.0%)	8 (17.0%)	5 (11.1%)	.09
Secondary or more	30 (78.9%)	40 (97.6%)	33 (91.7%)	40 (93.0%)	39 (83.0%)	40 (88.9%)	
No condom use in last 10 sexual encounters	22 (57.9%)	33 (80.5%)	28 (77.8%)	32 (74.4%)	32 (68.1%)	29 (64.4%)	.23
Typical frequency of intercourse (per mo)	13.3 ± 6.8	16.1 ± 6.5	13.9 ± 6.3	14.0 ± 6.5	15.3 ± 8.4	10.3 ± 7.2	.004^‡^
Sexually transmitted infections at screening							
*Chlamydia trachomatis*	5 (13.2%)	0	2 (5.6%)	4 (9.3%)	1 (2.1%)	3 (6.7%)	.11
*Neisseria gonorrhoeae*	1 (2.6%)	1 (2.4%)	1 (2.8%)	3 (7.0%)	0	0	.27
*Trichomonas vaginalis*	4 (10.5%)	1 (2.4%)	5 (13.9%)	3 (7.0%)	0	3 (6.7%)	.07
Using hormonal contraception at screening visit (not enrollment)	27 (71.1%)	29 (70.7%)	29 (80.6%)	30 (69.8%)	30 (63.8%)	25 (55.6%)	.27

Note

Data presented as mean ± SD or n (%) unless otherwise noted.

Abbreviations: Cu‐IUD, copper intrauterine device; DMPA, depot medroxyprogesterone acetate; ENG‐I, etonogestrel implant; IQR, interquartile range; LNG‐I, levonorgestrel implant; MPA/EC, medroxyprogesterone acetate and estradiol cypionate; Net‐En, norethisterone enanthate.

^†^ *P*‐value from Fisher's exact test unless otherwise noted.

^‡^ *P*‐value from one‐way analysis of variance.

^§^ *P*‐value from Kruskal‐Wallis test.

### 3.2 Serum progestin concentrations with contraceptive use

**Figure 2:**
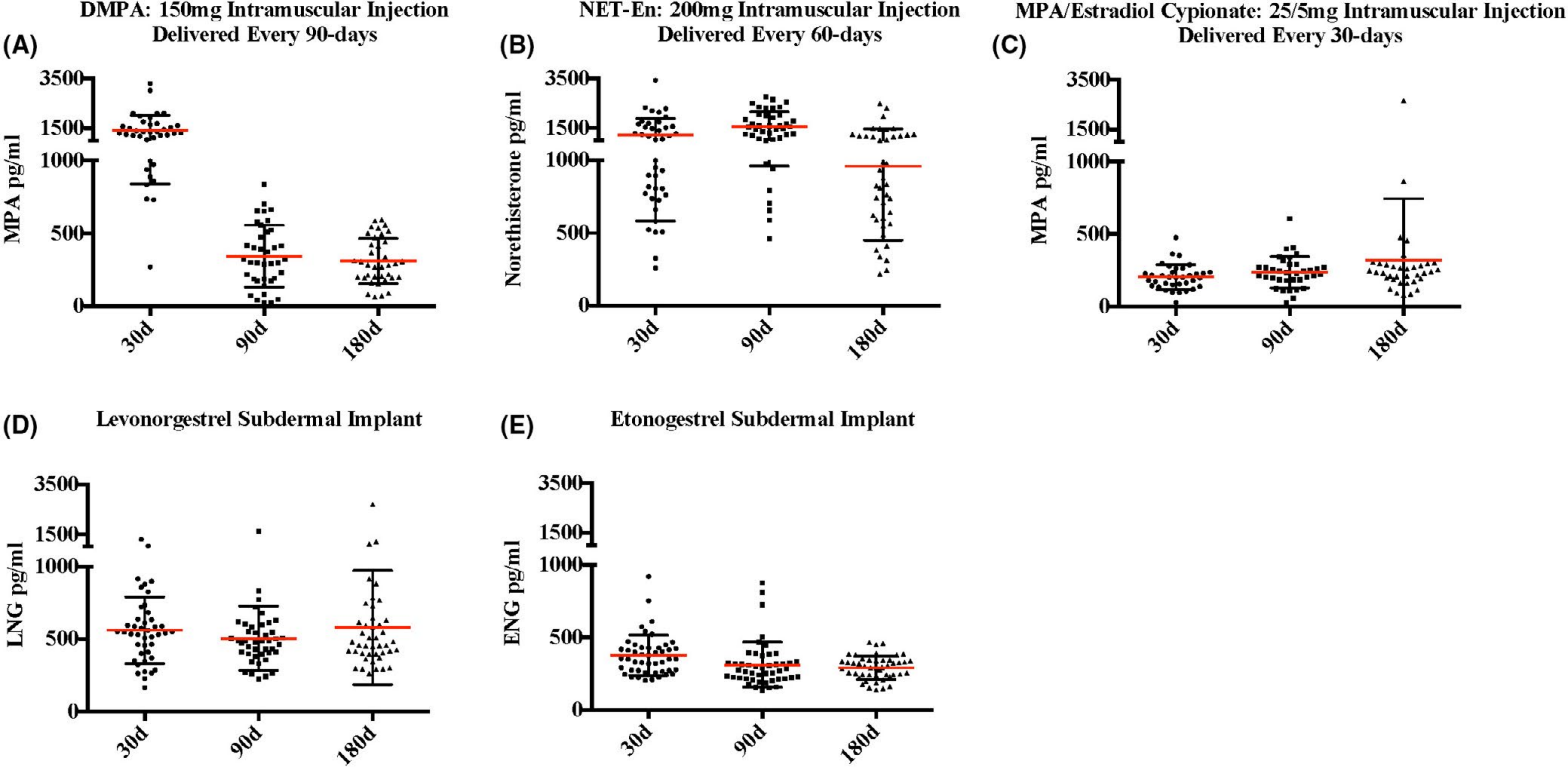
Systemic progestin concentrations after initiation and use of injectable and implantable contraceptives. Serum progestin concentrations for all evaluable participants at day 30, 90, and 180 following initiation and continuous use of (A) DMPA, (B) Net‐En, (C) MPA/estradiol cypionate, (D) levonorgestrel implant, and (E) etonogestrel implant. Mean serum concentrations with SD are indicated with red bars and black brackets, respectively. DMPA, depot medroxyprogesterone acetate; Net‐En, norethisterone enanthate; MPA, medroxyprogesterone acetate; LNG, levonorgestrel; ENG, etonogestrel

Figure 2 shows serum progestin concentrations for evaluable participants at day 30, 90, and 180 following hormonal contraceptive initiation. Given the varied clinical dosing schedules for the three injectable contraceptives, nadir serum concentrations (occurring immediately prior to re‐dosing) occur at day 90 and 180 in DMPA users (mean MPA = 343.8 and 311.7 pg/mL respectively vs 1428.2 pg/mL 30 days after dosing), day 180 in Net‐En users (mean NET = 960.8 pg/mL vs 1230.2 and 1562.6 pg/mL, respectively, at days 30 and 90, each 30 days after dosing), and every visit in MPA/EC users (mean MPA = 204.6, 238.1 and 320.1 pg/mL, respectively, at days 30, 90, and 180). Contraceptive implant users had relatively consistent serum progestin concentrations (mean LNG = 560.3, 505.8, and 578.8 pg/mL and mean ENG = 376.3, 311.1, and 290.9 pg/mL at days 30, 90, and 180 in LNG‐I and ENG‐I users, respectively).

### 3.3 Flow cytometry gating and baseline HIV target cell populations

**Figure 3:**
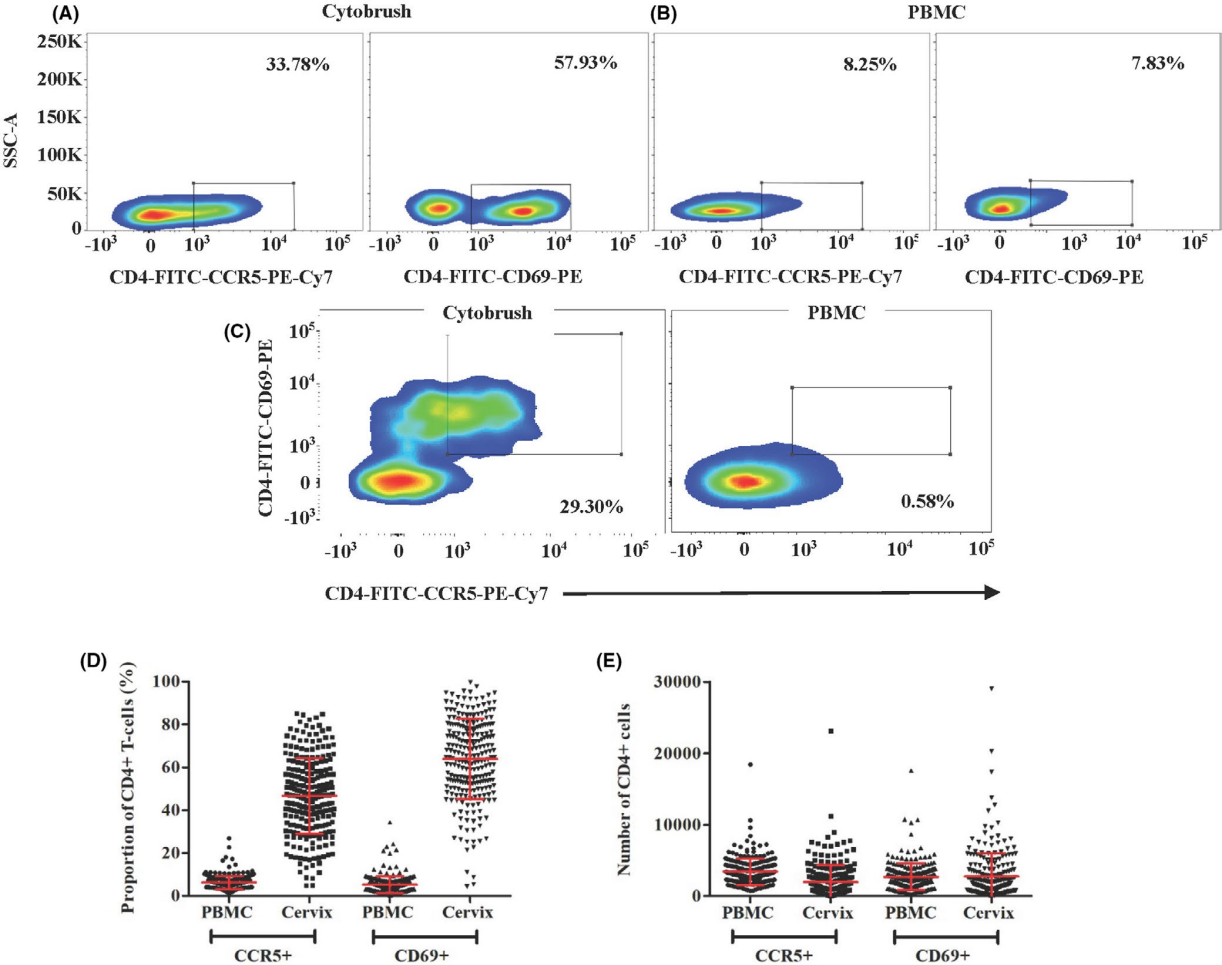
Flow cytometric gating strategy and baseline blood and cervical CD4 cells expressing CCR5 and CD69. Recovery of CD3^+^CD4^+^CCR5^+^ and CD3^+^CD4^+^CD69^+^ cells from (A) cervical cytobrush, (B) peripheral blood mononuclear cells (PBMC), (C) CD3^+^CD4^+^CCR5CD69^+^ double positives from cervical cytobrushes and PBMC (D) the proportion of CD3^+^CD4^+^CCR5^+^ and CD3^+^CD4^+^CD69^+^ from PBMC and cervical cytobrush demonstrating gating. Two senior laboratory scientists trained in advanced flow cytometry and masked to contraceptive group, independently reviewed and agreed upon gating parameters for each sample. The percent (D) and number (E) of CD3^+^CD4^+^CCR5^+^ and CD3^+^CD4^+^CD69^+^ cells in PBMC and cervical cytobrush samples collected from all evaluable participants at baseline, prior to initiation of any contraception. Mean values with SD are indicated with red bars and brackets, respectively

Live single CD3^+^ T cells were identified from the lymphocyte population based on forward and side scatter. The flow cytometric gating strategy for CD3^+^CD4^+^CCR5^+^ and CD3^+^CD4^+^CD69^+^ cellular populations in cytobrush and PBMC samples as well as the percent and number of CD3CD4CCR5^+^ and CD3CD4CD69^+^ cells in PBMC and cytobrush samples at enrollment prior to contraceptive initiation for all evaluable participants are shown in Figure 3. Live single CD11c^+^ cells were identified from the neutrophil, macrophage, dendritic cell populations based on forward and side scatter. Quantified median cell viabilities for PBMCs and endocervical cells were 99% and 72%, respectively.

### 3.4 Impact of injectable contraceptives

**Figure 4:**
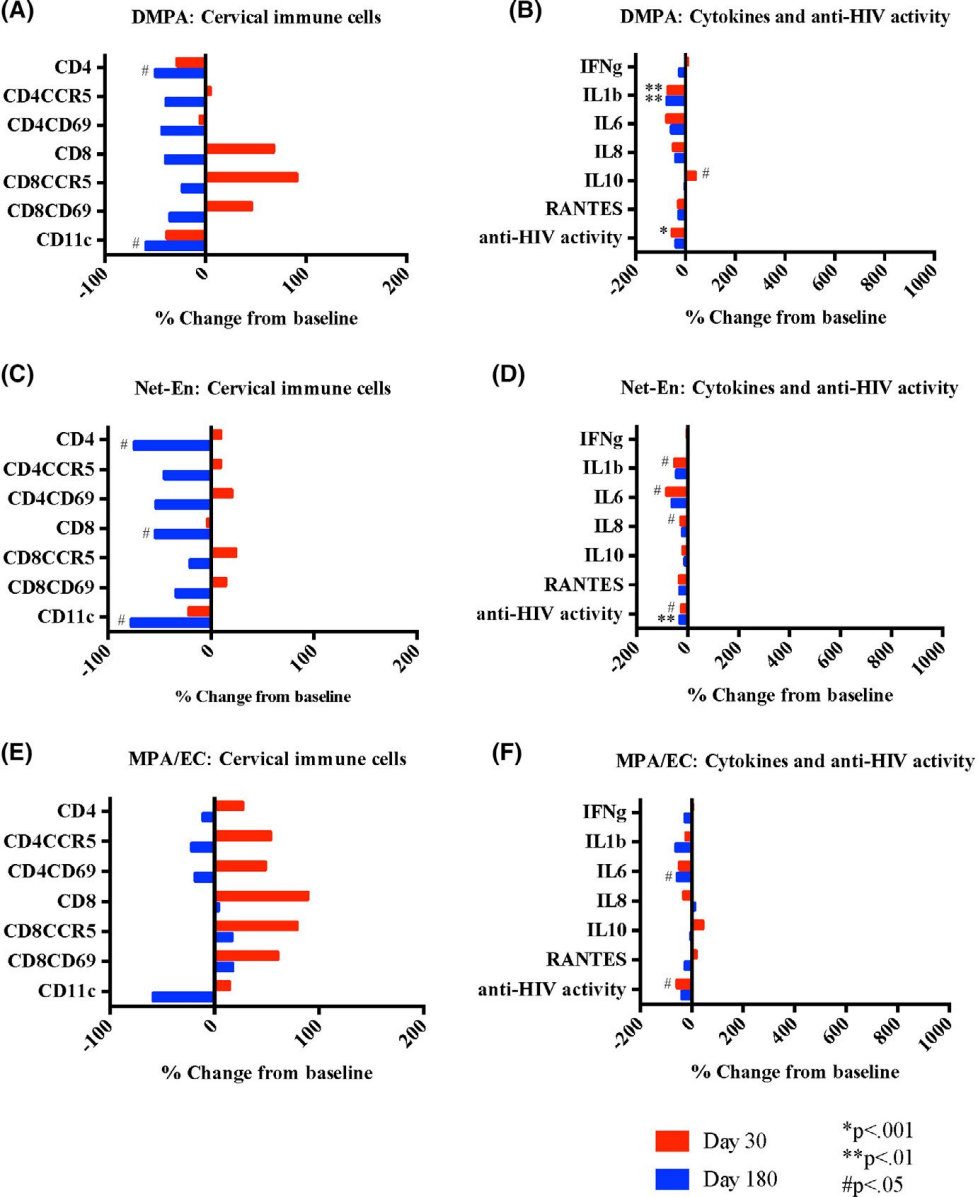
Impact of injectable contraceptives on genital immune cells and soluble mediators. Cervical immune cells (A, C, and E) collected by endocervical cytobrush and quantified by flow cytometry are expressed as % change in cell number from baseline to follow‐up at days 30 and 180 after initiation of depot medroxyprogesterone acetate (DMPA), norethisterone enanthate (Net‐En), or medroxyprogesterone acetate and estradiol cypionate (MPA/EC) as indicated. Soluble mediators and innate anti‐HIV activity (B, D, and F) measured in cervicovaginal lavage expressed as the % change from baseline to follow‐up at days 30 and 180 after injectable contraceptive initiation. *P*‐values from Wilcoxon signed‐rank test comparing baseline (prior to contraceptive initiation) to follow‐up 180 d after initiation and continuous use of contraceptive as indicated; *P*‐values adjusted using the Holm‐Bonferroni multiple test procedure

After initiating DMPA, most HIV target cells evaluated did not change in number (#) or proportion (%) compared with baseline. There were fewer %CD3CD4^+^ cells 30 days after and fewer #CD3CD4^+^ cells 180 days in the cervix after DMPA initiation (*P* < .001 and *P* = .04 respectively). The #CD11c^+^ APCs also decreased 180 days following DMPA initiation (*P* = .04) (Figure 4A and Table 2). In cervicovaginal fluid, IL‐1β decreased (*P* = .005 and *P* = .003) and there was a non‐significant trend toward decreased IL‐8 (*P* = .054 and *P* = .051) at 30 and 180 days after DMPA initiation. IL‐10 increased at day 30 (*P* = .03) and returned to baseline at day 180. There were no changes in IFN‐γ, IL‐6, or RANTES. Innate in vitro cervicovaginal anti‐HIV activity significantly decreased at day 30 (*P* < .001) (Figure 4B).

**Table 2:** Median cervical HIV target cells (# and %) at each study visit

	Baseline	30 d	*P*‐value	90 d	*P*‐value	180 d	*P*‐value
DMPA (n = 38)				^a^		^a^	
CD3CD4 #	3501 (1462, 8797)	3079 (1787, 4614)	.63	2570 (1525, 4889)	>.99	1777 (638, 3792)	.04
% (of CD3)	59.9 (51.0, 66.3)	47.5 (40.1, 55.6)	<.001	56.2 (46.8, 67.2)	>.99	55.6 (48.3, 61.6)	.16
CD3CD4CCR5 #	1431 (732, 3282)	1735 (635, 2576)	.77	1130 (781, 2870)	>.99	842 (246, 1968)	.22
% (of CD4)	43.3 (32.5, 55.1)	52.7 (43.1, 63.9)	.19	52.9 (41.0, 64.5)	.33	49.2 (35.7, 59.3)	.75
CD3CD4CD69 #	2189 (961, 5890)	2144 (1114, 3323)	.64	1910 (1030, 3940)	>.99	1207 (485, 2760)	.13
% (of CD4)	66.4 (53.3, 79.7)	72.4 (61.8, 83.4)	.35	77.1 (66.2, 84.7)	.38	77.1 (63.4, 86.9)	.28
CD11c #	41 423 (14 630, 92 730)	23 630 (12 394, 47 489)	.12	27 474 (10 968, 94 481)	.91	20 148 (83 121, 40 097)	.04
% (total cells)	21.5 (10.4, 34.1)	18.7 (7.1, 26.7)	.37	20.7(11.1, 31.1)	.96	11.3 (7.3, 20.8)	.13
Net‐En (n = 41)						^a^	
CD3CD4 #	2445 (1314, 7464)	2752 (1510, 4444)	>.99	2943 (1437, 6054)	.82	1473 (770, 2890)	.02
% (of CD3)	57.1 (46.6, 63.4)	53.1 (46.4, 61.2)	.80	57.7 (46.6, 64.2)	.99	53.1 (49.5, 64.4)	.49
CD3CD4CCR5 #	1030 (522, 2694)	1186 (548, 2442)	.81	1534 (792, 3200)	.33	815 (375, 1530)	.24
% (of CD4)	41.4 (31.4, 54.5)	50.5 (40.2, 59.3)	.18	54.9 (44.9, 67.3)	.01	59.7 (51.6, 70.7)	.001
CD3CD4CD69 #	1534 (808, 3252)	1773 (897, 3608)	>.99	1877 (1012, 4620)	>.99	1046 (545, 1992)	.10
% (of CD4)	63.1 (52.1, 76.2)	73.1 (62.2, 82.6)	.23	73.5 (63.6, 81.9)	.07	81.3 (63.7, 86.8)	.008
CD11c #	46 913 (15 530, 71 454)	29 592 (15 380, 60 116)	>.99	42 042 (12 794, 95 564)	>.99	12 719 (4598, 45 728)	.03
% (total cells)	22.2 (8.8, 29.3)	18.8 (11.5, 27.8)	.75	23.1 (12.9, 32.0)	.96	8.6 (4.3, 22.0)	.10
MPA/EC (n = 36)		^a^		^a^		^a^	
CD3CD4 #	2997 (656, 4609)	3541 (1535, 5376)	.63	3280 (1107, 4926)	>.99	1703 (958, 4942)	0.99
% (of CD3)	58.3 (45.0, 77.0)	55.1 (45.5, 64.7)	.60	53.1 (47.4, 63.5)	>.99	49.8 (42.1, 59.6)	.055
CD3CD4CCR5 #	1070 (412, 2543)	1578 (578, 2567)	.39	1177 (474, 2535)	0.97	1026 (476, 1815)	>.99
% (of CD4)	47.8 (37.0, 64.3)	43.2 (34.5, 59.7)	.56	45.7 (36.9, 58.5)	.83	48.5 (38.1, 61.7)	.84
CD3CD4CD69 #	1606 (585, 3149)	2437 (838, 3474)	.46	1886 (879, 3106)	>.99	1239 (685, 2505)	0.78
% (of CD4)	61.4 (47.4, 79.4)	70.3 (55.6, 83.0)	>.99	68.2 (51.3, 80.3)	>.99	74.3 (50.1, 82.1)	>.99
CD11c #	32 348 (15 788, 93 308)	62 569 (14 388, 117 058)	.64	39 300 (12 721, 104 467)	>.99	11 032 (6046, 30 469)	0.33
% (total cells)	13.9 (7.9, 26.3)	22.9 (12.9, 35.9)	.19	18.1 (10.8, 32.9)	>.99	9.6 (6.1, 21.1)	.65
LNG implant (n = 43)							
CD3CD4 #	3750 (1620, 6259)	3703 (1499, 8590)	>.99	3298 (1438, 6129)	>.99	2467 (956, 5536)	>.99
% (of CD3)	55.5 (43.9, 66.2)	53.2 (42.7, 59.9)	.79	59.2 (50.6, 66.2)	>.99	57.6 (48.5, 63.8)	>.99
CD3CD4CCR5 #	1176 (701, 3053)	1255 (646, 2447)	>.99	1235 (457, 2692)	>.99	1011 (376, 3118)	>.99
% (of CD4)	50.6 (31.5, 63.0)	43.0 (29.1, 56.2)	.77	39.2 (28.2, 60.7)	>.99	44.9 (34.0, 55.8)	>.99
CD3CD4CD69#	2044 (1000, 3713)	2441 (961, 4623)	>.99	1908 (859, 3873)	>.99	1518 (698, 4167)	>.99
% (of CD4)	61.9 (51.1, 80.5)	67.4 (54.8, 80.4)	>.99	68.9 (47.3, 78.4)	>.99	71.1 (53.2, 81.8)	>.99
CD11c #	51 638 (19 500, 109 558)	66 993 (16 091, 112 285)	>.99	41 476 (16 810, 103 974)	>.99	49 895 (17 002, 128 656)	.88
% (total cells)	27.9 (14.2, 37.3)	26.8 (13.6, 39.6)	.91	22.4 (12.1, 37.8)	>.99	23.8 (8.1, 37.1)	>.99
ENG implant (n = 47)							
CD3CD4 #	2621 (1036, 7400)	2780 (1451, 5287)	>.99	1748 (1087, 3856)	>.99	1591 (832, 3829)	.19
% (of CD3)	58.5 (44.3, 67.1)	53.4 (45.9, 63.6)	.70	55.4 (42.1, 65.3)	>.99	53.2 (46.4, 60.0)	.19
CD3CD4CCR5 #	1447 (591, 2296)	1115 (572, 2110)	>.99	893 (556, 1931)	>.99	899 (349, 1834)	.60
% (of CD4)	49.3 (32.9, 62.1)	42.7 (34.0, 58.9)	.98	50.8 (39.6, 63.0)	>.99	49.1 (37.1, 64.3)	.76
CD3CD4CD69 #	1610 (823, 3598)	1829 (828, 3739)	>.99	1351 (775, 2278)	>.99	1260 (670, 2336)	.82
% (of CD4)	70.4 (52.9, 82.1)	68.6 (54.6, 80.4)	>.99	76.6 (60.7, 88.6)	.16	76.0 (66.1, 87.4)	.050
CD11c #	36 591 (14 019, 86 085)	32 111 (11 105, 70 577)	>.99	24 300	.99	10 959 (5317, 41 668)	.002
% (total cells)	21.1 (11.2, 33.6)	19.1 (8.6, 28.1)	.84	17.3 (9.6, 28.5)	>.99	12.9 (7.1, 21.2)	.004
Cu‐IUD (n = 45)							
CD3CD4 #	2028 (872, 5229)	5539 (1985, 10 728)	<.001	3791 (1710, 9873)	.09	3179 (1442, 6696)	.58
% (of CD3)	56.1 (46.5, 65.9)	60.9 (50.7, 65.5)	.51	59.2 (49.3, 64.6)	.76	58.0 (51.1, 64.2)	>.99
CD3CD4CCR5 #	875 (305, 2449)	1385 (903, 3314)	.02	1643 (822, 4536)	.16	1184 (547, 1988)	>.99
% (of CD4)	47.8 (39.6, 57.7)	36.2 (26.5, 48.5)	.01	48.7 (36.7, 67.1)	.98	39.5 (29.5, 59.4)	.77
CD3CD4CD69 #	1211 (620, 2642)	3692 (1340, 6980)	<.001	2256 (1320, 6820)	.02	1422 (690, 3954)	.37
% (of CD4)	64.7 (53.2, 78.5)	64.8 (51.9, 71.2)	.85	72.2 (56.0, 81.7)	.54	66.9 (51.2, 79.7)	.75
CD11c #	32 806 (14 500, 67 872)	86 844 (45 266, 190 526)	.003	69 898 (44 494, 161 526)	.054	83 055 (21 842, 157 280)	.11
% (total cells)	18.5 (6.8, 27.2)	28.2 (16.3, 38.8)	.06	26.4 (20.2, 34.3)	.07	27.2 (11.5, 38.0)	.73

Note

Data are displayed as median (interquartile range). *P*‐values from Wilcoxon signed‐rank test comparing baseline (prior to contraceptive initiation) to follow‐up 30, 90, and 180 d after initiation and continuous use of contraceptive; *P*‐values adjusted using the Holm‐Bonferroni multiple test procedure.

Abbreviations: Cu‐IUD, copper intrauterine device; DMPA, depot medroxyprogesterone acetate; ENG, etonogestrel; LNG, levonorgestrel; MPA/EC, medroxyprogesterone acetate and estradiol cypionate; Net‐En, norethisterone enanthate.

^a^ Represents nadir serum concentration as sampling was immediately prior to next dosing.

Compared to baseline, women using Net‐En had no changes in any cervical immune cell populations evaluated after 30 days, had increased %CD4CCR5^+^ (*P* = .01) after 90 days, and after 180 days had decreased #CD3CD4^+^ (*P* = .02) and #CD11c^+^ (*P* = .03) cells and increased %CD4CCR5^+^ and %CD4CD69^+^ cells (*P* = .001 and *P* = .008, respectively) (Figure 4C and Table 2). In cervicovaginal fluid, IL‐1β, IL‐6, and IL‐8 decreased with Net‐En use (*P* = .04, *P* = .02, *P* = .03, respectively) at day 30 and returned to baseline by day 180 (Figure 4D). There were no changes in IFN‐γ, IL‐10, or RANTES concentrations. Innate in vitro cervicovaginal anti‐HIV activity significantly decreased at day 30 (*P* = .02) and day 180 (*P* = .001) after Net‐En initiation.

After initiating MPA/EC, women had no changes in any cellular populations evaluated at any time point (Figure 4E and Table 2). IL‐6 decreased at day 180 (*P* = .01) and there were no other changes in any of the soluble immune mediators evaluated (Figure 4F). Innate in vitro cervicovaginal anti‐HIV activity decreased transiently at day 30 (*P* = .04) after MPA/EC initiation.

### 3.5 Impact of contraceptive implants

**Figure 5:**
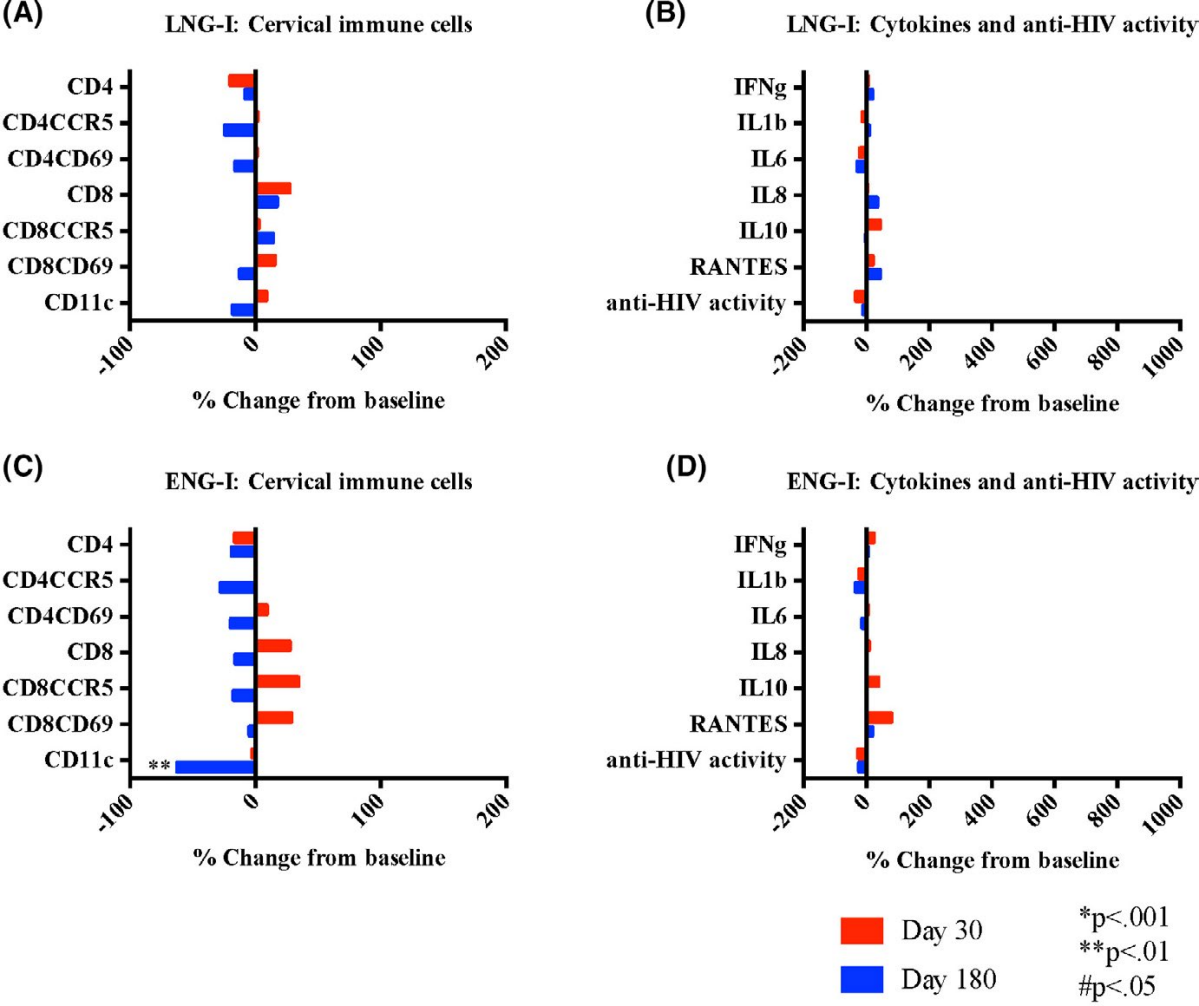
Impact of implantable contraceptives on genital immune cells and soluble mediators. Cervical immune cells (A and C) collected by endocervical cytobrush and quantified by flow cytometry are expressed as % change in cell number from baseline to follow‐up at days 30 and 180 after initiation of contraception with levonorgestrel implant (LNG‐I) or etonogestrel implant (ENG‐I) as indicated. Soluble mediators and innate anti‐HIV activity (B and D) measured in cervicovaginal lavage expressed as the % change from baseline to follow‐up at days 30 and 180 after implantable contraceptive initiation. *P*‐values from Wilcoxon signed‐rank test comparing baseline (prior to contraceptive initiation) to follow‐up 180 d after initiation and continuous use of contraceptive as indicated; *P*‐values adjusted using the Holm‐Bonferroni multiple test procedure

After initiating LNG‐I, there were no changes in cervical or systemic HIV target cell populations, cytokines and soluble mediators, or innate in vitro anti‐HIV activity (Figure 5A,B and Table 2).

Compared to baseline, there were no changes in any immune cell populations after 30 or 90 days of ENG‐I use, and decreased CD11c cells (*P* = .002 and *P* = .004 for # and %, respectively) after 180 days use (Figure 5C and Table 2). There were no changes in any of the soluble mediators evaluated or the innate in vitro cervicovaginal anti‐HIV activity through 180 days of use (Figure 5D).

### 3.6 Impact of copper intrauterine device

**Figure 6:**
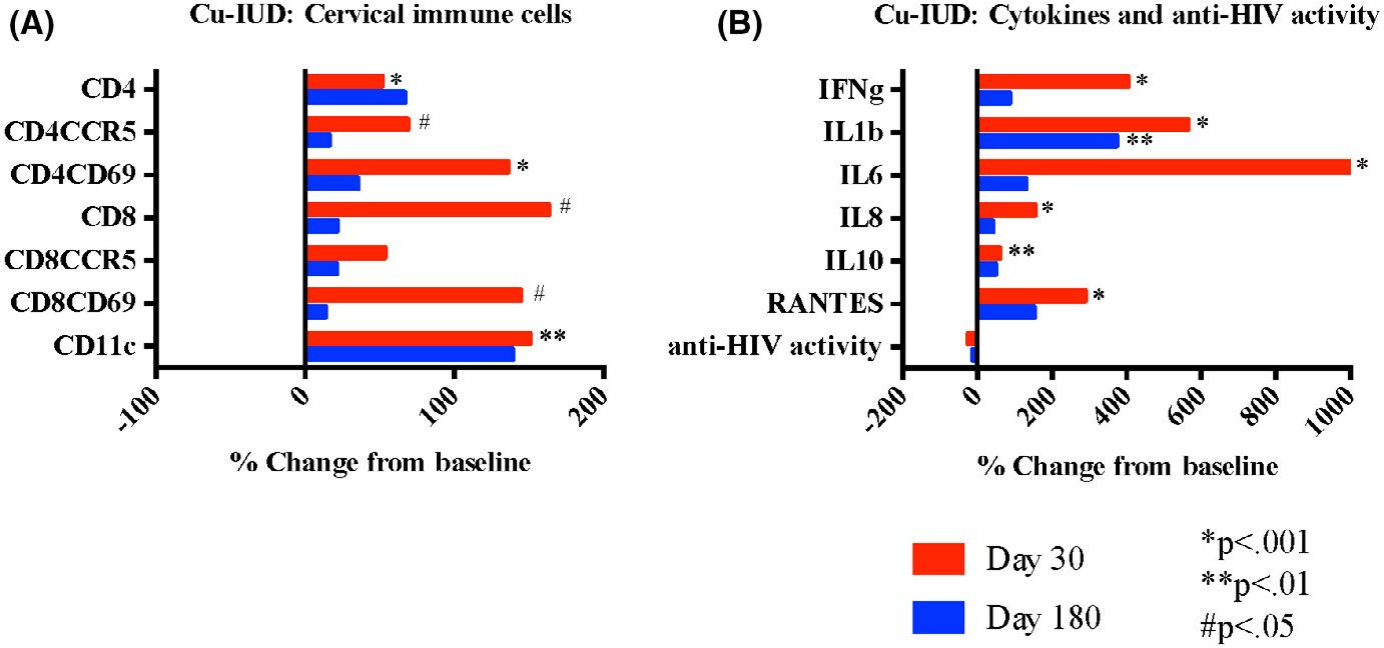
Impact of copper intrauterine device on genital immune cells and soluble mediators. Cervical immune cells (A) collected by endocervical cytobrush and quantified by flow cytometry are expressed as % change in cell number from baseline to follow‐up at days 30 and 180 after initiation of contraception with copper intrauterine device (Cu‐IUD). Soluble mediators and innate anti‐HIV activity (B) measured in cervicovaginal lavage expressed as the % change from baseline to follow‐up at days 30 and 180 after intrauterine contraceptive initiation. *P*‐values from Wilcoxon signed‐rank test comparing baseline (prior to contraceptive initiation) to follow‐up 180 d after initiation and continuous use of contraceptive as indicated; *P*‐values adjusted using the Holm‐Bonferroni multiple test procedure

Thirty days after initiating Cu‐IUD, we observed significant increases in most of the cervical HIV target cells evaluated, including #CD4^+^ (*P* < .001), #CD4CCR5^+^ (*P* = .02), #CD4CD69^+^ (*P* < .001), and #CD11c^+^ (*P* = .003), (Figure 6A and Table 2). The %CD4CCR5^+^ were decreased at 30 days (*P* = .01). By 180 days following Cu‐IUD insertion, all cell populations had returned to baseline. All soluble mediators were significantly increased 30 days after Cu‐IUD initiation (IFN‐γ *P* < .001, IL‐1β *P* < .001, IL‐6 *P* < .001, IL‐8 *P* < .001, IL‐10 *P* = .002, and RANTES *P* < .001), and resolved by day 180 except for sustained elevation in IL‐1β (*P* = .004) (Figure 6B). Innate in vitro cervicovaginal anti‐HIV activity did not significantly change with Cu‐IUD use. The results were similar when analyses were restricted to women who initiated Cu‐IUD and who were negative for BV at all visits (n = 21).

### 3.7 Impact of contraceptive use on cervical CD8 cells and PBMCs

There were no changes in cervical CD8 cell populations with initiation and use of MPA/EC, LNG‐I, or ENG‐I (Table S1). Compared to baseline, women using DMPA had increased %CD3^+^CD8^+^ cells in the cervix after 30 and 180 days (*P* = .008 and *P* = .04 respectively). Net‐En users had decreased #CD3CD8^+^ cells (*P* = .04) after 180 days, increased %CD8CCR5^+^ after 90 and 180 days (*P* = .003 and *P* < .001, respectively), and increased %CD8CD69^+^ (*P* = .009) after 180 days. Cu‐IUD users had transiently increased #CD8^+^ and #CD8CD69^+^ (*P* = .04 and *P* = .01, respectively) and decreased %CD8CCR5^+^ (*P* = .02) 30 days after insertion.

PBMC populations did not change in women using DMPA, MPA/EC, LNG‐I, or Cu‐IUD over 180 days and we observed isolated increases in systemic #CD8CCR5^+^ cells in Net‐En users at 90 days and #CD8CD69^+^ cells in ENG‐I users at 180 days (*P* = .009 and *P* = .025 respectively) (Table S2).

## 4 DISCUSSION

Given concerns that use of DMPA may increase HIV acquisition risk, we quantified biological changes within the reproductive tract associated with contraceptive initiation that could plausibly relate to HIV susceptibility. We focused on genital tract HIV target cell changes, soluble inflammatory mediators, and innate in vitro anti‐HIV activity following contraceptive initiation as have several other recent investigations.^25^, ^26^, ^27^, ^28^, ^29^, ^30^ We found that use of DMPA and other injectables were linked with modest changes after 6 months of use, predominantly decreasing HIV target cells and inflammatory mediators. We found minimal changes in cervical HIV target cells and inflammatory markers associated with use of hormonal contraceptive implants. In contrast, although the Cu‐IUD has often been used as a non‐hormonal comparator, our results show that the Cu‐IUD is not immunologically inert. We found that Cu‐IUD initiation was associated with biological changes that could, at least temporarily, alter HIV susceptibility, including marked increases in HIV target cells, marked increases in proinflammatory cytokines, and increased BV (previously reported),^20^ although the physiological relevance of these changes remains uncertain. In these analyses, BV is unlikely driving the observed cellular and cytokine changes.

Most of the changes, we observed with DMPA use would not be predicted to increase HIV acquisition. We did find that DMPA users had a measured transient decrease in innate in vitro anti‐HIV activity in cervicovaginal fluid at day 30 although the clinical significance of this finding is uncertain.^31^ We similarly observed this effect in users of Net‐En and MPA/EC. Hormonal effects may depend on the type and concentration of progestin administered.^32^, ^33^ It remains possible that peak serum DMPA concentrations may have associated immunologic alterations that could transiently increase HIV susceptibility, which may deserve further study.

Our results differ somewhat from other recently published data, including our own,^18^, ^25^, ^26^, ^27^, ^28^ in which investigators have reported changes in genital HIV target cells based solely on proportional changes. Notably, proportional data for cellular populations must be interpreted within the context of the actual number of cells. For instance, prior to conducting the multiple comparison adjustment, in women initiating DMPA our unadjusted data suggested a non‐significant decrease in the number of CD4 cells coupled with a non‐significant increase in CD4CCR5 cells at 30 days, which mathematically created a significant increase in %CD4 cells expressing CCR5. Further, since CD4CCR5^+^ cells are also quantified in the broader CD4 cell group, if there is a decrease in the parent group and the number of CD4CCR5^+^ cells remains the same, this results in a greater proportion of CD4CCR5^+^ cells, and not a greater number or density of HIV targets for infection. Furthermore, flow cytometry data become less stable with small numbers of cells, thus reporting numbers of recovered cells are particularly important for non‐blood samples where numbers may be low. Here, we have demonstrated robust numbers of cells collected by cervical cytobrush sampling, which is a strength of these data.

Given the historically low use of Cu‐IUD among women in high HIV prevalence areas, there are insufficient clinical data to assess if Cu‐IUD use alters risk of HIV acquisition. Cu‐IUD was included as a comparator arm in the recently published ECHO trial (NCT02550067),^4^ designed to compare HIV acquisition risk in women randomized to DMPA, LNG‐implant, and Cu‐IUD. In this trial, women randomized to Cu‐IUD and to DMPA had very similar HIV acquisition, whereas women randomized to LNG‐implant had a non‐significant trend toward lower HIV acquisition. Unlike ECHO, which was designed to assess HIV acquisition, our study was designed to assess biological changes associated with contraceptive initiation and use that may relate to HIV susceptibility. Like ECHO, our data support that contraceptive implants would unlikely alter HIV susceptibility given they appear inert with respect to HIV target cell populations and inflammation.

Our head‐to‐head study compared six contraceptive methods and included measurement of contraceptive progestin concentrations to ensure the biological exposure and to rule out exposure to other methods. Both contraceptives and biological mechanisms are complex to study in women and a major strength of this study is the rigorous measurement of hormonal milieu. Contraceptives vary with respect to progestin‐type, route of administration, and resultant serum drug concentrations. Further complicating contraceptive research is common reliance on self‐report, frequent contraceptive switching and intraindividual variability in progestin metabolism, thus confirming hormonal exposure is critical to data interpretation. We found that analyzing data at time points of relatively high and low (nadir) progestin concentrations were more informative than time since initiation.

Our study has some limitations, including self‐selection of contraception and limited evaluation of estrogen‐containing contraceptives, with the sole combined hormonal method (MPA/EC) having unfortunate timing of dosing such that study samples were only collected at nadir hormone concentrations. Similarly, we were unable to characterize immune changes during true peak serum hormone concentrations as no samples were collected earlier than 30 days following contraceptive initiation. Finally, since there are no known reliable biomarkers for HIV risk, we selected HIV target cells and soluble mediators of interest based on hypothesized biologic mechanisms. We acknowledge that only a small subset of cellular and soluble mediators was studied and there are many other potential biologic mechanisms that were not investigated here.

Provision of effective contraception to all women seeking to avoid pregnancy remains a global health priority. DMPA initiation and use do not markedly alter HIV target cell populations and soluble immune mediators in the genital tract and Net‐En initiation appears immunologically similar to DMPA. The copper IUD is not inert with respect to genital tract inflammation as measured by marked increases in cellular and functional immune activity and this may be mainly associated with insertion given our findings of resolution of most effects by 90 days. Contraceptive implant use appears inert given low and stable serum progestin concentration and minimally altered genital tract HIV target cells and immune mediators.

## CONFLICT OF INTEREST

The funder had no role in study design, data collection, data analysis, data interpretation, or manuscript writing. The corresponding author had full access to all study data and final responsibility for the decision to submit for publication. SLA has served as a consultant to Merck Sharp & Dohme Corp and has received research grants from Mithra and Evofem. SLH is a consultant for Merck, Pfizer, Lupin, Hologic, Pfizer, and Daré Bioscience, and receives research funding from Cepheid, Becton‐Dickinson, and Curatek. All other authors declare no competing interests.

## AUTHOR CONTRIBUTIONS

SLA designed the study protocol with assistance from LAM and SLH. FGM and ZMC oversaw the clinical research site and team and collected participant samples. Laboratory processing of samples, running of assays, and flow cytometry was conducted and overseen by ATM, KAS, and MAB. LAM was responsible for database management. SLA, SLH, and LAM analyzed and interpreted the data. SLA wrote the manuscript. All authors have read and commented on the final report.

## IMPLICATIONS

Although the copper IUD has often been used as a non‐hormonal comparator, we show that it is not immunologically inert, particularly in the first 30 days following insertion, which is an important framework for data interpretation. Future work focused on investigating biological mechanisms underlying the observed contraceptive‐induced immune alterations may benefit future contraceptive and multipurpose product development.

## DISCLAIMER

The protocol for this clinical study is publicly available at https://mageewomens.org/investigator/sharon‐achilles‐md‐phd/. The de‐identified data that support the findings of this study are available upon request from the corresponding author [SLA]. The data are not publicly available due to containing information that could compromise research participant privacy/consent.

## Supporting information

TableS1‐S2

## References

[1] United Nations. Department of Economic and Social Affairs, Population Division . Trends in Contraceptive Use Worldwide 2015 (ST/ESA/SER.A/349). 2015.

[2] Birth Control Around the World: Mapping Methods of Contraception | Superdrug^™^ . https://onlinedoctor.superdrug.com/birth‐control‐around‐the‐world/. Accessed February 15, 2019

[3] Polis CB , Curtis KM , Hannaford PC , et al. An updated systematic review of epidemiological evidence on hormonal contraceptive methods and HIV acquisition in women. AIDS. 2016;30(17):2665‐2683. 27500670 10.1097/QAD.0000000000001228 PMC5106090

[4] Evidence for Contraceptive Options and HIV Outcomes (ECHO) Trial Consortium . HIV incidence among women using intramuscular depot medroxyprogesterone acetate, a copper intrauterine device, or a levonorgestrel implant for contraception: a randomised, multicentre, open‐label trial. Lancet. 2019;394(10195):303‐313. 31204114 10.1016/S0140-6736(19)31288-7 PMC6675739

[5] Gosmann C , Anahtar MN , Handley SA , et al. Lactobacillus‐deficient cervicovaginal bacterial communities are associated with increased HIV acquisition in young South African Women. Immunity. 2017;46(1):29‐37. 28087240 10.1016/j.immuni.2016.12.013 PMC5270628

[6] McClelland RS , Lingappa JR , Srinivasan S , et al. Evaluation of the association between the concentrations of key vaginal bacteria and the increased risk of HIV acquisition in African women from five cohorts: a nested case‐control study. Lancet Infect Dis. 2018;18(5):554‐564. 29396006 10.1016/S1473-3099(18)30058-6 PMC6445552

[7] Eastment MC , McClelland RS . Vaginal microbiota and susceptibility to HIV. AIDS. 2018;32(6):687‐698. 29424773 10.1097/QAD.0000000000001768 PMC5957511

[8] Cohen CR , Lingappa JR , Baeten JM , et al. Bacterial vaginosis associated with increased risk of female‐to‐male HIV‐1 transmission: a prospective cohort analysis among African couples. PLoS Medicine. 2012;9(6):e1001251. 22745608 10.1371/journal.pmed.1001251 PMC3383741

[9] Farcasanu M , Kwon DS . The influence of cervicovaginal microbiota on mucosal immunity and prophylaxis in the battle against HIV. Curr HIV/AIDS Rep. 2018;15(1):30‐38. 29516267 10.1007/s11904-018-0380-5

[10] Cohen CR , Moscicki A‐B , Scott ME , et al. Increased levels of immune activation in the genital tract of healthy young women from sub‐Saharan Africa. AIDS. 2010;24(13):2069‐2074. 20588163 10.1097/QAD.0b013e32833c323b PMC2914808

[11] Masson L , Passmore J‐A , Liebenberg LJ , et al. Genital inflammation and the risk of HIV acquisition in women. Clin Infect Dis. 2015;61(2):260‐269. 25900168 10.1093/cid/civ298 PMC4565995

[12] Kaul R , Prodger J , Joag V , et al. Inflammation and HIV transmission in sub‐saharan Africa. Curr HIV/AIDS Rep. 2015;12(2):216‐222. 25877253 10.1007/s11904-015-0269-5

[13] Schacker T , Little S , Connick E , et al. Productive infection of T cells in lymphoid tissues during primary and early human immunodeficiency virus infection. J Infect Dis. 2001;183(4):555‐562. 11170980 10.1086/318524

[14] Loré K , Smed‐Sörensen A , Vasudevan J , Mascola JR , Koup RA . Myeloid and plasmacytoid dendritic cells transfer HIV‐1 preferentially to antigen‐specific CD4+ T cells. J Exp Med. 2005;201(12):2023‐2033. 15967828 10.1084/jem.20042413 PMC2212038

[15] Moore JP , Trkola A , Dragic T . Co‐receptors for HIV‐1 entry. Curr Opin Immunol. 1997;9(4):551‐562. 9287172 10.1016/s0952-7915(97)80110-0

[16] Achilles SL , Mhlanga FG , Musara P , Poloyac SM , Chirenje ZM , Hillier SL . Misreporting of contraceptive hormone use in clinical research participants. Contraception. 2018;97(4):346‐353. 28966052 10.1016/j.contraception.2017.09.013 PMC5858917

[17] Zhang J , Tang C , Oberly PJ , Minnigh MB , Achilles SL , Poloyac SM . A sensitive and robust UPLC–MS/MS method for quantitation of estrogens and progestogens in human serum. Contraception. 2019;99(4):244‐250. 30685285 10.1016/j.contraception.2018.12.010 PMC6441366

[18] Achilles SL , Creinin MD , Stoner KA , Chen BA , Meyn L , Hillier SL . Changes in genital tract immune cell populations after initiation of intrauterine contraception. Am J Obstet Gynecol. 2014;211(5):489.e1‐489.e9. 24834865 10.1016/j.ajog.2014.05.016 PMC4231025

[19] Study of Immune Cell Changes in the Genital Tract 2 Months After Initiation of an IUD for Contraception ‐ Full Text View ‐ ClinicalTrials.gov. https://clinicaltrials.gov/ct2/show/NCT01240811. Accessed September 12, 2018

[20] Achilles SL , Austin MN , Meyn LA , Mhlanga F , Chirenje ZM , Hillier SL . Impact of contraceptive initiation on vaginal microbiota. Am J Obstet Gynecol. 2018;218(6):622.e1‐622.e10. 29505773 10.1016/j.ajog.2018.02.017 PMC5990849

[21] Strober W . Trypan blue exclusion test of cell viability. Curr Protoc Immunol. 2001;Appendix 3:Appendix 3B 10.1002/0471142735.ima03bs21 18432654

[22] Nugent RP , Krohn MA , Hillier SL . Reliability of diagnosing bacterial vaginosis is improved by a standardized method of gram stain interpretation. J Clin Microbiol. 1991;29(2):297‐301. 1706728 10.1128/jcm.29.2.297-301.1991 PMC269757

[23] Dezzutti CS , Hendrix CW , Marrazzo JM , et al. Performance of swabs, lavage, and diluents to quantify biomarkers of female genital tract soluble mucosal mediators. PLoS One. 2011;6(8):e23136. 21858008 10.1371/journal.pone.0023136 PMC3155537

[24] Holm S . A simple sequentially rejective multiple test procedure. Scand J Stat. 1979;6(2):65‐70.

[25] Li L , Zhou J , Wang W , et al. Effects of three long‐acting reversible contraceptive methods on HIV target cells in the human uterine cervix and peripheral blood. Reprod Biol Endocrinol. 2019;17. 10.1186/s12958-019-0469-8 PMC6387540 30795774

[26] Lajoie J , Tjernlund A , Omollo K , et al. Increased cervical CD4+CCR5+ T cells among kenyan sex working women using depot medroxyprogesterone acetate. AIDS Res Hum Retroviruses. 2019;35(3):236‐246. 30585733 10.1089/aid.2018.0188 PMC6434599

[27] Byrne EH , Anahtar MN , Cohen KE , et al. Association between injectable progestin‐only contraceptives and HIV acquisition and HIV target cell frequency in the female genital tract in South African women: a prospective cohort study. Lancet Infect Dis. 2016;16(4):441‐448. 26723758 10.1016/S1473-3099(15)00429-6 PMC5294917

[28] Smith‐McCune KK , Hilton JF , Shanmugasundaram U , et al. Effects of depot‐medroxyprogesterone acetate on the immune microenvironment of the human cervix and endometrium: implications for HIV susceptibility. Mucosal Immunol. 2017;10(5):1270‐1278. 28051087 10.1038/mi.2016.121 PMC5496803

[29] Mitchell CM , McLemore L , Westerberg K , et al. Long‐term effect of depot medroxyprogesterone acetate on vaginal microbiota, epithelial thickness and HIV target cells. J Infect Dis. 2014;210(4):651‐655. 24652495 10.1093/infdis/jiu176 PMC4172039

[30] Sciaranghella G , Wang C , Hu H , et al. CCR5 expression levels in HIV‐uninfected women receiving hormonal contraception. J Infect Dis. 2015;212(9):1397‐1401. 25895986 10.1093/infdis/jiv233 PMC4601918

[31] Levinson P , Kaul R , Kimani J , et al. Levels of innate immune factors in genital fluids: association of alpha defensins and LL‐37 with genital infections and increased HIV acquisition. AIDS. 2009;23(3):309‐317. 19114868 10.1097/QAD.0b013e328321809c

[32] Polis CB , Achilles SL , Hel Z , Hapgood JP . Is a lower‐dose, subcutaneous contraceptive injectable containing depot medroxyprogesterone acetate likely to impact women’s risk of HIV? Contraception. 2018;97(3):191‐197. 29242082 10.1016/j.contraception.2017.12.003 PMC5828886

[33] Heffron R , Achilles SL , Dorflinger LJ , et al. Pharmacokinetic, biologic and epidemiologic differences in MPA‐ and NET‐based progestin‐only injectable contraceptives relative to the potential impact on HIV acquisition in women. Contraception. 2019;99(4):199‐204. 30576636 10.1016/j.contraception.2018.12.001 PMC6467541

