# Supplementary material for: Zim CHIC: A cohort study of immune changes in the female genital tract associated with initiation and use of contraceptives
Source: Am J Reprod Immunol. 2020 Jun 25;84(3):e13287. doi: 10.1111/aji.13287 (PMC7507197; doi:10.1111/aji.13287)
Supplement: Supplementary file 1 — TableS1‐S2 [file AJI-84-e13287-s001.docx]

**Table A.1. Median cervical CD8 cells (# and %) at each study visit**

|  | Baseline | 30 days | p-value | 90 days | p-value | 180 days | p-value |
| --- | --- | --- | --- | --- | --- | --- | --- |
| DMPA (n=38) |  |  |  | ***** |  | ***** |  |
| CD8 # | 1809 (891, 33558) | 2570 (1265, 4996) | .67 | 1215 (777, 3377) | >.99 | 1100 (527, 2706) | .21 |
| % (of CD3) | 32.5 (25.2, 40.4) | 44.2 (33.1, 49.8) | .008 | 30.7 (25.2, 46.3) | >.99 | 38.9 (32.4, 49.8) | .04 |
| CD8CCR5 # | 907 (525, 1656) | 1556 (477, 3116) | .78 | 696 (405, 1698) | >.99 | 565 (255, 1725) | .26 |
| % (of CD4) | 54.6 (41.9, 70.3) | 57.8 (37.9, 71.3) | .69 | 53.0 (40.2, 72.8) | >.99 | 54.4 (39.8, 68.0) | >.99 |
| CD8CD69 # | 1459 (663, 2706) | 2052 (821, 3798) | .84 | 1040 (692, 2575) | >.99 | 926 (390, 1830) | .28 |
| % (of CD4) | 80.2 (71.7, 90.0) | 80.1 (64.5, 88.3) | >.99 | 85.7 (75.3, 92.6) | >.99 | 80.2 (70.2, 91.1) | .88 |
| Net-En (n=41) |  |  |  |  |  | ***** |  |
| CD8 # | 1525 (828, 4230) | 1900 (1077, 3079) | >.99 | 1696 (1190, 3732) | >.99 | 855 (602, 1571) | .04 |
| % (of CD3) | 35.3 (31.0, 42.8) | 39.3 (30.1, 46.2) | .33 | 35.8 (23.6, 43.6) | .77 | 42.7 (31.0, 46.9) | .27 |
| CD8CCR5 # | 819 (278, 1604) | 1052 (415, 1864) | >.99 | 1238 (564, 1960) | .42 | 617 (336, 1080) | .29 |
| % (of CD4) | 49.9 (33.1, 56.6) | 52.5 (42.4, 61.5) | .31 | 63.5 (46.6, 79.2) | .003 | 69.2 (53.3, 81.6) | <.001 |
| CD8CD69 # | 1194 (600, 2474) | 1205 (682, 2464) | >.99 | 1377 (780, 2862) | .79 | 734 (524, 1282) | .12 |
| % (of CD4) | 76.1 (64.2, 85.8) | 83.3 (65.7, 87.6) | .88 | 82.4 (71.8, 87.4) | .24 | 87.6 (77.7, 92.3) | .009 |
| MPA/EC (n=36) |  | ***** |  | ***** |  | ***** |  |
| CD8 # | 1328 (515, 2166) | 2023 (1190, 3614) | .15 | 1620 (911, 3548) | >.99 | 1364 (668, 4247) | >.99 |
| % (of CD3) | 37.9 (23.5, 46.7) | 38.1 (28.9, 46.0) | .91 | 38.7 (29.6, 44.4) | >.99 | 42.4 (33.7, 49.2) | .56 |
| CD8CCR5 # | 640 (276, 1013) | 986 (614, 1476) | .77 | 857 (366, 2072) | >.99 | 722 (448, 1767) | >.99 |
| % (of CD4) | 52.8 (40.4, 70.7) | 45.0 (36.4, 64.1) | .20 | 52.8 (38.9, 73.0) | >.99 | 55.2 (48.9, 73.4) | >.99 |
| CD8CD69 # | 820 (403, 1448) | 1531 (822, 2238) | .20 | 1264 (634, 2331) | >.99 | 1068 (566, 2162) | >.99 |
| % (of CD4) | 81.2 (58.4, 92.0) | 81.7 (52.7, 89.4) | .81 | 78.3 (60.4, 89.1) | >.99 | 78.4 (60.3, 87.7) | >.99 |
| LNG implant (n=43) |  |  |  |  |  |  |  |
| CD8 # | 2471 (854, 4723) | 1882 (748, 5600) | >.99 | 1687 (978, 2748) | >.99 | 1565 (568, 3758) | >.99 |
| % (of CD3) | 33.5 (26.2, 47.2) | 36.2 (26.2, 46.7) | >.99 | 30.5 (27.3, 41.4) | .93 | 35.9 (26.1, 40.7) | >.99 |
| CD8CCR5 # | 882 (472, 2451) | 651 (363, 1895) | .99 | 635 (374, 1530) | >.99 | 726 (256, 2023) | >.99 |
| % (of CD4) | 54.7 (38.8, 74.3) | 45.2 (33.3, 60.6) | .11 | 47.7 (32.9, 56.5) | >.99 | 50.1 (39.0, 64.7) | >.99 |
| CD8CD69 # | 1655 (570, 3517) | 1478 (659, 3550) | >.99 | 1171 (775, 2065) | >.99 | 1291 (516, 2733) | >.99 |
| % (of CD4) | 78.8 (65.0, 88.2) | 77.8 (63.8, 87.5) | >.99 | 78.8 (64.3, 89.7) | >.99 | 81.5 (68.8, 89.1) | >.99 |
| ENG implant (n=47) |  |  |  |  |  |  |  |
| CD8 # | 1424 (563, 3589) | 2018 (1208, 3173) | >.99 | 1388 (647, 2388) | >.99 | 1271 (662, 2429) | .66 |
| % (of CD3) | 36.6 (25.6, 42.6) | 42.2 (29.4, 47.4) | .27 | 37.2 (25.6, 47.9) | >.99 | 41.5 (32.4, 50.9) | .12 |
| CD8CCR5 # | 916 (340, 1652) | 972 (374, 1655) | .97 | 703 (269, 1499) | .87 | 746 (368, 1432) | .53 |
| % (of CD4) | 55.8 (38.0, 70.6) | 53.4 (34.9, 62.1) | .67 | 55.5 (44.3, 70.4) | .98 | 56.2 (45.1, 75.2) | .62 |
| CD8CD69 # | 1115 (407, 2395) | 1349 (640, 2301) | >.99 | 1100 (505, 1846) | >.99 | 999 (547, 1706) | >.99 |
| % (of CD4) | 79.8 (71.7, 90.1) | 79.8 (65.7, 88.2) | .82 | 87.4 (69.7, 93.0) | .47 | 86.1 (74.4, 91.7) | .28 |
| Cu-IUD (n=45) |  |  |  |  |  |  |  |
| CD8 # | 1519 (416, 3482) | 2542 (1177, 5192) | .04 | 1908 (898, 3030) | >.99 | 1765 (776, 3856) | >.99 |
| % (of CD3) | 39.7 (25.6, 49.1) | 31.9 (24.7, 38.1) | .10 | 33.2 (19.7, 36.6) | .08 | 35.3 (25.6, 41.5) | .92 |
| CD8CCR5 # | 597 (176, 2106) | 754 (538, 2072) | .35 | 924 (468, 1588) | .92 | 734 (356, 1710) | .93 |
| % (of CD4) | 54.9 (41.4, 64.9) | 38.4 (25.1, 54.7) | .02 | 52.3 (37.8, 67.7) | >.99 | 46.8 (32.7, 63.6) | .83 |
| CD8CD69 # | 944 (293, 2184) | 1830 (932, 4010) | .01 | 1292 (780, 2532) | >.99 | 1130 (55, 2972) | >.99 |
| % (of CD4) | 74.8 (65.4, 84.5) | 77.3 (62.9, 82.8) | >.99 | 80.8 (67.3, 89.5) | .61 | 76.2 (62.1, 87.5) | >.99 |

Data are displayed as median (interquartile range)

* Represents nadir serum concentration as sampling was immediately prior to next dosing

DMPA=depot medroxyprogesterone acetate; Net-En=norethisterone enanthate; MPA/EC=medroxyprogesterone acetate and estradiol cypionate; LNG=levonorgestrel; ENG=etonogestrel; Cu-IUD=copper intrauterine device

*P*-values from Wilcoxon signed-rank test comparing baseline (prior to contraceptive initiation) to follow-up 30, 90, and 180 days after initiation and continuous use of contraceptive; *P*-values adjusted using the Holm-Bonferroni multiple test procedure

**Table A.2. Median peripheral blood mononuclear cell (PBMC) numbers (#) at each study visit**

|  | Baseline | 30 days | p-value | 90 days | p-value | 180 days | p-value |
| --- | --- | --- | --- | --- | --- | --- | --- |
| DMPA (n=38) |  |  |  | ***** |  | ***** |  |
| CD4 | 55581 (49721, 64445) | 56928 (49250, 64284) | >.99 | 56188 (48824, 65563) | >.99 | 56718 (50656, 66024) | >.99 |
| CD4CCR5 | 2888 (2060, 3644) | 3038 (2326, 4235) | >.99 | 3136 (2387, 4666) | .65 | 2912 (1908, 4192) | >.99 |
| CD4CD69 | 2555 (1644, 3872) | 3054 (1838, 3802) | >.99 | 2508 (1923, 4473) | .82 | 2127 (1493, 2861) | >.99 |
| CD8 | 29313 (22929, 34668) | 28465 (23429, 33450) | .93 | 30374 (23342, 35100) | >.99 | 29380 (23432, 33774) | .79 |
| CD8CCR5 | 2785 (2062, 4014) | 2510 (2034, 4622) | >.99 | 3552 (1976, 4984) | >.99 | 2776 (1850, 4575) | >.99 |
| CD8CD69 | 1854 (1300, 2281) | 1814 (1349, 2294) | >.99 | 2129 (1208, 2650) | >.99 | 1353 (1226, 1860) | >.99 |
| CD11c | 12408 (8428, 14630) | 13016 (10062, 15585) | >.99 | 11316 (8626, 18609) | >.99 | 9486 (8338, 11905) | >.99 |
| Net-En (n=41) |  |  |  |  |  | ***** |  |
| CD4 | 54024 (49216, 61084) | 58074 (50761, 62623) | >.99 | 54852 (46645, 60930) | >.99 | 54747 (47789, 58690) | >.99 |
| CD4CCR5 | 3019 (1918, 4264) | 2548 (1640, 3594) | .87 | 3570 (2654, 4570) | .26 | 2953 (2652, 3774) | .83 |
| CD4CD69 | 2197 (1378, 3370) | 2563 (1634, 4066) | >.99 | 2634 (1766, 3770) | >.99 | 2384 (1576, 3198) | >.99 |
| CD8 | 29714 (24330, 34729) | 30713 (23822, 34364) | .93 | 30076 (24032, 35006) | >.99 | 29086 (25214, 33808) | >.99 |
| CD8CCR5 | 2928 (1922, 3906) | 2450 (1881, 4176) | >.99 | 3664 (2318, 5362) | .009 | 3740 (2638, 4572) | .06 |
| CD8CD69 | 1664 (1249, 2106) | 1812 (1170, 2251) | >.99 | 1764 (1188, 2281) | >.99 | 1636 (1426, 2197) | .66 |
| CD11c | 13985 (8310, 19256) | 14666 (10920, 22088) | .28 | 11850 (8803, 19622) | .87 | 11092 (7201, 14130) | .08 |
| MPA/EC (n=36) |  | ***** |  | ***** |  | ***** |  |
| CD4 | 55000 (49969, 59934) | 53959 (45856, 62216) | .90 | 56352 (49243, 61210) | >.99 | 56894 (50372, 62070) | >.99 |
| CD4CCR5 | 3219 (2336, 4510) | 2816 (1300, 4331) | .96 | 3370 (2486, 4572) | .75 | 3496 (2700, 4118) | .89 |
| CD4CD69 | 2296 (1453, 3285) | 3082 (1983, 3874) | .91 | 1924 (1457, 2712) | >.99 | 2020 (1475, 3178) | >.99 |
| CD8 | 30018 (24196, 33659) | 31130 (25078, 36558) | .94 | 29409 (24222, 33846) | >.99 | 29094 (24438, 33230) | >.99 |
| CD8CCR5 | 2747 (2072, 4678) | 3202 (1788, 4797) | >.99 | 2738 (2144, 5116) | >.99 | 2780 (2226, 4834) | >.99 |
| CD8CD69 | 1615 (1095, 2124) | 1872 (1330, 2360) | .91 | 1585 (1181, 2266) | >.99 | 1524 (1274, 2059) | >.99 |
| CD11c | 13370 (7088, 18826) | 13753 (9322, 19296) | .94 | 11091 (8757, 17123) | >.99 | 9274 (6852, 17264) | .67 |
| LNG implant (n=43) |  |  |  |  |  |  |  |
| CD4 | 53583 (45628, 59613) | 56187 (46524, 60489) | >.99 | 53543 (46586, 59319) | .87 | 54158 (48889, 63814) | >.99 |
| CD4CCR5 | 2929 (2205, 3738) | 2445 (1682, 4054) | .32 | 2857 (2219, 3981) | >.99 | 3466 (1922, 4615) | >.99 |
| CD4CD69 | 2367 (1453, 3349) | 2885 (2140, 3417) | .61 | 2205 (1202, 3089) | >.99 | 2497 (1508, 3248) | >.99 |
| CD8 | 31593 (27420, 36499) | 33057 (28408, 37845) | >.99 | 30369 (25346, 34928) | .23 | 34624 (27431, 36704) | >.99 |
| CD8CCR5 | 3479 (2239, 6279) | 3065 (2301, 5755) | .99 | 3799 (2243, 5622) | >.99 | 3637 (2769, 5711) | >.99 |
| CD8CD69 | 1488 (919, 2065) | 1677 (1188, 2445) | .12 | 1559 (990, 2371) | >.99 | 1681 (1241, 2794) | .053 |
| CD11c | 10334 (7387, 17619) | 10777 (7976, 14860) | .76 | 11263 (9210, 16758) | >.99 | 10320 (8575, 16342) | .97 |
| ENG implant (n=47) |  |  |  |  |  |  |  |
| CD4 | 55828 (49705, 63821) | 55364 (48181, 62718) | >.99 | 57619 (46632, 62185) | >.99 | 59182 (48980, 62662) | >.99 |
| CD4CCR5 | 3480 (2651, 4303) | 2856 (1932, 4591) | >.99 | 3474 (2132, 5461) | >.99 | 3658 (2583, 4727) | .69 |
| CD4CD69 | 2248 (1690, 3220) | 2495 (1373, 3193) | .99 | 2765 (1632, 3498) | .53 | 2409 (1829, 3376) | >.99 |
| CD8 | 27872 (22499, 32907) | 28431 (23497, 34670) | >.99 | 29447 (22413, 34416) | .84 | 27756 (23166, 32714) | >.99 |
| CD8CCR5 | 3121 (2133, 4235) | 3335 (2212, 5164) | >.99 | 3990 (2093, 5706) | .67 | 3107 (2302, 4873) | >.99 |
| CD8CD69 | 1568 (1004, 1929) | 1349 (995, 1910) | >.99 | 1418 (1030, 2021) | >.99 | 1621 (1245, 2281) | .025 |
| CD11c | 12683 (7940, 15867) | 12656 (7225, 17409) | >.99 | 13641 (9506, 19230) | >.99 | 11796 (9507, 17346) | >.99 |
| Cu-IUD (n=45) |  |  |  |  |  |  |  |
| CD4 | 56627 (49617, 63030) | 55896 (50466, 63742) | >.99 | 57427 (49958, 63048) | >.99 | 57370 (51671, 61607) | >.99 |
| CD4CCR5 | 3092 (2170, 4126) | 3113 (2122, 4113) | >.99 | 3257 (2394, 4444) | .85 | 3066 (2670, 4246) | >.99 |
| CD4CD69 | 2585 (2010, 3358) | 2664 (1590, 3598) | >.99 | 2811 (1588, 3987) | >.99 | 2316 (1802, 3515) | >.99 |
| CD8 | 29397 (25454, 35680) | 31540 (25281, 37672) | .67 | 29014 (24834, 35191) | >.99 | 29361 (24562, 33290) | .26 |
| CD8CCR5 | 2879 (2354, 3960) | 3285 (2215, 4460) | >.99 | 2916 (2252, 5194) | >.99 | 3529 (2520, 2072) | >.99 |
| CD8CD69 | 1487 (1270, 2042) | 1769 (1258, 2352) | >.99 | 1781 (1158, 2033) | >.99 | 1685 (1166, 2072) | .91 |
| CD11c | 13844 (9311, 17612) | 14470 (9886, 19940) | .96 | 12885 (9720, 21975) | >.99 | 11498 (8394, 16570) | .86 |

Data are displayed as median (interquartile range)

*Represents nadir serum concentration as sampling was immediately prior to next dosing

DMPA=depot medroxyprogesterone acetate; Net-En=norethisterone enanthate; MPA/EC=medroxyprogesterone acetate and estradiol cypionate; LNG=levonorgestrel; ENG=etonogestrel; Cu-IUD=copper intrauterine device

*P*-values from Wilcoxon signed-rank test comparing baseline (prior to contraceptive initiation) to follow-up 30, 90, and 180 days after initiation and continuous use of contraceptive; *P*-values adjusted using the Holm-Bonferroni multiple test procedure
